# 2-Iodo-*N*-(2-nitro­phenyl­sulfan­yl)aniline

**DOI:** 10.1107/S1600536808019491

**Published:** 2008-07-05

**Authors:** Iván Brito, Alejandro Cárdenas, Aldo Mundaca, Matías López-Rodríguez, Andrea Reyes

**Affiliations:** a Departamento de Química, Facultad de Ciencias Básicas, Universidad de Antofagasta, Casilla 170, Antofagasta, Chile; b Departamento de Física, Facultad de Ciencias Básicas, Universidad de Antofagasta, Casilla 170, Antofagasta, Chile; c Instituto de Bio-Orgánica ’Antonio González’, Universidad de La Laguna, Astrofísico Francisco Sánchez N°2, La Laguna, Tenerife, Spain

## Abstract

In title compound, C_12_H_9_IN_2_O_2_S, the nitro group is rotated slightly, by 8.91 (3)°, out of the plane of the aromatic ring to which it is bonded. Between the two aromatic rings the CSN plane is at a dihedral angle of 84.0 (7)° to the HNC plane. Mol­ecules are linked by C—H⋯O inter­actions into a double helical supra­molecular architecture. There are no iodo–nitro, π–π or C—H⋯π(arene) inter­actions.

## Related literature

For related literature, see: Bernstein *et al.* (1995 ▶); Brito *et al.* (2004 ▶, 2005 ▶, 2006 ▶); Glidewell *et al.* (2003 ▶); Kuhle (1973 ▶); Pauling (1960 ▶).
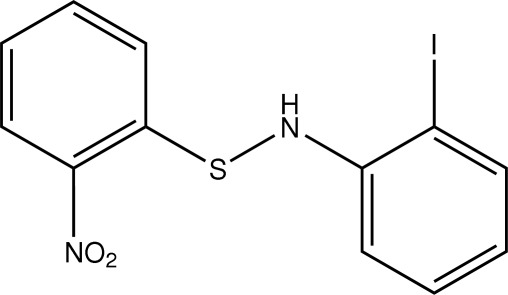


## Experimental

### 

#### Crystal data

C_12_H_9_IN_2_O_2_S
*M* *_r_* = 372.17
Trigonal, 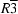

*a* = 28.6221 (12) Å
*c* = 8.4062 (17) Å
*V* = 5963.9 (13) Å^3^
*Z* = 18
Mo *K*α radiation
μ = 2.57 mm^−1^
*T* = 298 (2) K
0.47 × 0.32 × 0.20 mm

#### Data collection

Nonius KappaCCD area-detector diffractometer
Absorption correction: multi-scan (*SORTAV*; Blessing, 1995 ▶) *T*_min_ = 0.380, *T*_max_ = 0.600
11358 measured reflections
3251 independent reflections
2801 reflections with *I* > 2σ(*I*)
*R*_int_ = 0.059

#### Refinement

*R*[*F* ^2^ > 2σ(*F* ^2^)] = 0.072
*wR*(*F* ^2^) = 0.205
*S* = 1.21
3251 reflections
166 parameters
H-atom parameters constrained
Δρ_max_ = 1.04 e Å^−3^
Δρ_min_ = −1.05 e Å^−3^

### 

Data collection: *COLLECT* (Nonius, 2000 ▶); cell refinement: *DENZO-SMN* (Otwinowski & Minor, 1997 ▶); data reduction: *DENZO-SMN*; program(s) used to solve structure: *SIR97* (Altomare *et al.*, 1999 ▶); program(s) used to refine structure: *SHELXL97* (Sheldrick, 2008 ▶); molecular graphics: *ORTEP-3 for Windows* (Farrugia, 1997 ▶) and *PLATON* (Spek, 2003 ▶); software used to prepare material for publication: *WinGX* (Farrugia, 1999 ▶).

## Supplementary Material

Crystal structure: contains datablocks global, I. DOI: 10.1107/S1600536808019491/fl2204sup1.cif

Structure factors: contains datablocks I. DOI: 10.1107/S1600536808019491/fl2204Isup2.hkl

Additional supplementary materials:  crystallographic information; 3D view; checkCIF report

## Figures and Tables

**Table 1:** Hydrogen-bond geometry (Å, °)

*D*—H⋯*A*	*D*—H	H⋯*A*	*D*⋯*A*	*D*—H⋯*A*
C10—H10⋯O1^i^	0.93	2.55	3.445 (10)	161

Symmetry code: (i) 
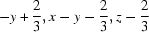
.

**Fig. 1.**
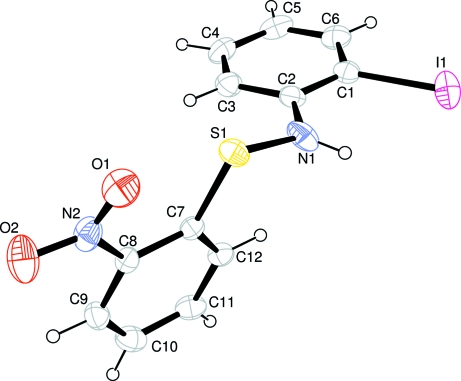
The molecule of compound (I), showing the atom-labelling scheme. Displacement ellipsoids are drawn at the 30% probability level and H atoms are shown as small spheres of arbitrary radii.

**Fig. 2.**
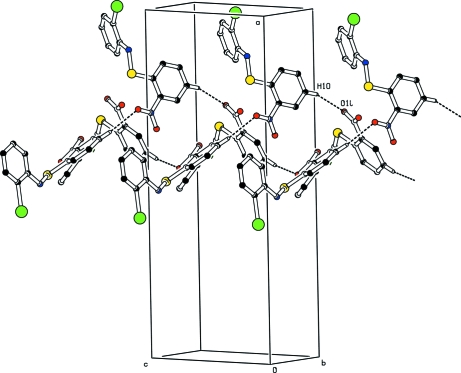
A portio nof the packing diagram for (I), showing the double helical arrangement of molecules along [001]. For the sake of clarity, H atoms not involved in the motif shown have been omitted. [Symmetry code: (i) 2/3 - y, -2/3 + x-y, -2/3 + z]

## supplementary crystallographic information

### Comment

Sulfenamides are important compounds with versatile industrial applications (Kuhle, 1973). Bond polarization in sulfenamide derivatives, resulting from the difference in electronegativity between sulfur and nitrogen, activates the S—N bond for attack by both nucleophiles and electrophiles, and appears to be the factor primarily responsible for the chemistry of these compounds. The title compound, (I), is a positional isomer of 4-Iodo -*N*-(2-nitrophenylsulfanyl)-aniline (Glidewell *et al.*,2003) and shows excellent agreement with its bonding geometries. The title compound is the result of the condensation reaction of 2-nitrophenylsulfenyl choride and 1-iodoaniline. Its structure is described here as part of our work involving the study of the synthesis and structural characterization of divalent-sulfur compounds (Brito *et al.*, 2004, 2005, 2006). A view of the molecular structure of (I) is given in Fig.1. In (I) the 1-Iodo-benzene fragment is connected by an –NH—S– linker unit to the 2-nitrophenyl fragment. It contains an N atom as a chiral center, though the material is a racemic mixture. The nitro group is rotated by 8.91 (3)°. The S—N distance of 1.696 (6)Å is shorter than a normal S—N single-bond length (1.74 Å, Pauling, 1960), but is normal for this type of structure, many of which have S—N single bonds in the range 1.63–1.68 Å as a result of the π character of the S—N bond. The C7/S1/N1 plane makes a dihedral angle of 84.0 (7) ° with the H1/N1/C2 plane, in good agreement with the values of ~90.0 ° for the torsional ground state of this type species. The molecules are linked into a double helical supramolecular architecture with only hydrogen bonding contributing to the double helical arrangement (Bernstein *et al.*, 1995). Atom C10 and nitro atom O1 in the molecule at (*x*, *y*, *z*) act as hydrogen-bond donor and acceptor respectively, Fig.2, Table 1. There are no iodo-nitro, π-π and C—H··· π (arene) interactions.

### Experimental

A sample of compound (I) was prepared by reaction of equimolar quantities of 2-nitrophenylsulfenyl chloride (0.01 mol,1.895 g) and 4-iodoaniline (0.01 mol, 2.190 g) in dichloromethane solution, in the presence of an excess of triethylamine. Purification was by thin-layer chromatography and crystals of (I) suitable for single-crystal X-ray diffraction were grown by slow evaporation of a solution in ethanol [m.p. 472 K]. FT—IR (KBr pellet, cm^-1^): ν (w, N—H amine) 3091, ν (w, S—N) 1039, ν (w, C—S) 731, ν (s, C—H disubstitution) 855, ν (*versus*, NO_2_) 1567.

### Refinement

All H atoms were initially located in a difference Fourier map and were subsequently refined using a riding model, with C—H distances of 0.93 Å and *U*_iso_(H)= 1.2 *U*_eq_(C) for benzene H atoms and N—H = 0.86 Å for amino H atom and *U*_iso_(H) = 1.2*U*_eq_(N).

### Crystal data

**Table:**

C_12_H_9_IN_2_O_2_S	*Z* = 18
*M**_r_* = 372.17	*F*_000_ = 3240
Trigonal, *R*3	*D*_x_ = 1.865 Mg m^−^^3^
Hall symbol: -R 3	Melting point: 472 K
*a* = 28.6221 (12) Å	Mo *K*α radiation λ = 0.71073 Å
*b* = 28.6221 (12) Å	Cell parameters from 909 reflections
*c* = 8.4062 (17) Å	θ = 1.4–28.5º
α = 90º	µ = 2.57 mm^−^^1^
β = 90º	*T* = 298 (2) K
γ = 120º	Prism, yellow
*V* = 5963.9 (13) Å^3^	0.47 × 0.32 × 0.20 mm

### Data collection

**Table:**

Nonius KappaCCD area-detector diffractometer	2801 reflections with *I* > 2σ(*I*)
Radiation source: fine-focus sealed tube	*R*_int_ = 0.059
Monochromator: graphite	θ_max_ = 28.5º
φ scans, and ω scans with κ offsets	θ_min_ = 1.4º
Absorption correction: multi-scan(SORTAV; Blessing, 1995)	*h* = −31→37
*T*_min_ = 0.380, *T*_max_ = 0.600	*k* = −36→35
11358 measured reflections	*l* = −7→11
3251 independent reflections	

### Refinement

**Table:**

Refinement on *F*^2^	H-atom parameters constrained
Least-squares matrix: full	*w* = 1/[σ^2^(*F*_o_^2^) + (0.087*P*)^2^ + 39.1076*P*] where *P* = (*F*_o_^2^ + 2*F*_c_^2^)/3
*R*[*F*^2^ > 2σ(*F*^2^)] = 0.072	(Δ/σ)_max_ = 0.001
*wR*(*F*^2^) = 0.205	Δρ_max_ = 1.04 e Å^−^^3^
*S* = 1.21	Δρ_min_ = −1.05 e Å^−^^3^
3251 reflections	Extinction correction: SHELXL97 (Sheldrick, 2008), Fc^*^=kFc[1+0.001xFc^2^λ^3^/sin(2θ)]^-1/4^
166 parameters	Extinction coefficient: 0.0064 (7)

### Special details

**Table:**

Geometry. All e.s.d.'s (except the e.s.d. in the dihedral angle between two l.s. planes) are estimated using the full covariance matrix. The cell e.s.d.'s are taken into account individually in the estimation of e.s.d.'s in distances, angles and torsion angles; correlations between e.s.d.'s in cell parameters are only used when they are defined by crystal symmetry. An approximate (isotropic) treatment of cell e.s.d.'s is used for estimating e.s.d.'s involving l.s. planes.
Refinement. Refinement of *F*^2^ against ALL reflections. The weighted *R*-factor *wR* and goodness of fit *S* are based on *F*^2^, conventional *R*-factors *R* are based on *F*, with *F* set to zero for negative *F*^2^. The threshold expression of *F*^2^ > σ(*F*^2^) is used only for calculating *R*-factors(gt) *etc*. and is not relevant to the choice of reflections for refinement. *R*-factors based on *F*^2^ are statistically about twice as large as those based on *F*, and *R*- factors based on ALL data will be even larger.

### Fractional atomic coordinates and isotropic or equivalent isotropic displacement parameters (Å^2^)

**Table:**

	*x*	*y*	*z*	*U*_iso_*/*U*_eq_	
I1	1.003222 (18)	0.07997 (2)	0.27240 (7)	0.0680 (3)	
S1	0.81458 (6)	−0.00495 (6)	0.12352 (17)	0.0458 (4)	
N1	0.8810 (2)	0.0367 (2)	0.1625 (7)	0.0565 (13)	
H1	0.9042	0.0289	0.1244	0.068*	
N2	0.70588 (19)	−0.0418 (2)	−0.0484 (7)	0.0529 (12)	
O1	0.7146 (2)	−0.0679 (2)	0.0488 (7)	0.0679 (13)	
O2	0.6610 (2)	−0.0547 (3)	−0.0985 (9)	0.0865 (19)	
C1	0.9522 (2)	0.1110 (2)	0.3204 (7)	0.0450 (12)	
C2	0.8998 (2)	0.0847 (2)	0.2574 (6)	0.0427 (11)	
C3	0.8677 (3)	0.1073 (3)	0.2903 (7)	0.0498 (13)	
H3	0.8328	0.0909	0.25	0.06*	
C4	0.8862 (3)	0.1532 (3)	0.3809 (8)	0.0559 (15)	
H4	0.8642	0.1679	0.3997	0.067*	
C5	0.9383 (3)	0.1777 (3)	0.4448 (8)	0.0596 (17)	
H5	0.9507	0.2083	0.5081	0.072*	
C6	0.9706 (3)	0.1569 (2)	0.4143 (7)	0.0518 (14)	
H6	1.0053	0.1733	0.4562	0.062*	
C7	0.80300 (19)	0.02629 (18)	−0.0433 (6)	0.0340 (9)	
C8	0.75198 (19)	0.00701 (19)	−0.1113 (6)	0.0365 (10)	
C9	0.7438 (2)	0.0326 (3)	−0.2401 (7)	0.0482 (13)	
H9	0.7093	0.0191	−0.2816	0.058*	
C10	0.7861 (3)	0.0775 (3)	−0.3060 (7)	0.0549 (15)	
H10	0.7809	0.0938	−0.3946	0.066*	
C11	0.8368 (3)	0.0982 (2)	−0.2383 (7)	0.0495 (13)	
H11	0.8656	0.1294	−0.2798	0.059*	
C12	0.8450 (2)	0.0729 (2)	−0.1094 (6)	0.0391 (10)	
H12	0.8795	0.0875	−0.0663	0.047*	

### Atomic displacement parameters (Å^2^)

**Table:**

	*U*^11^	*U*^22^	*U*^33^	*U*^12^	*U*^13^	*U*^23^
I1	0.0502 (3)	0.0627 (4)	0.0973 (5)	0.0329 (2)	−0.0026 (2)	0.0077 (2)
S1	0.0515 (8)	0.0395 (7)	0.0445 (7)	0.0213 (6)	−0.0044 (6)	0.0046 (5)
N1	0.060 (3)	0.049 (3)	0.057 (3)	0.025 (2)	−0.028 (3)	−0.012 (2)
N2	0.033 (2)	0.045 (3)	0.067 (3)	0.009 (2)	0.004 (2)	−0.008 (2)
O1	0.055 (3)	0.052 (3)	0.076 (3)	0.011 (2)	0.014 (2)	0.021 (2)
O2	0.036 (2)	0.075 (4)	0.124 (5)	0.009 (2)	−0.009 (3)	−0.004 (3)
C1	0.052 (3)	0.046 (3)	0.038 (3)	0.025 (2)	0.004 (2)	0.011 (2)
C2	0.057 (3)	0.046 (3)	0.031 (2)	0.030 (2)	−0.004 (2)	−0.001 (2)
C3	0.059 (3)	0.054 (3)	0.044 (3)	0.034 (3)	−0.001 (2)	−0.002 (2)
C4	0.066 (4)	0.060 (4)	0.050 (3)	0.037 (3)	0.014 (3)	−0.003 (3)
C5	0.073 (4)	0.048 (3)	0.044 (3)	0.020 (3)	0.015 (3)	−0.003 (2)
C6	0.052 (3)	0.045 (3)	0.044 (3)	0.013 (2)	0.006 (2)	0.006 (2)
C7	0.036 (2)	0.030 (2)	0.034 (2)	0.0157 (18)	0.0034 (18)	−0.0011 (17)
C8	0.032 (2)	0.033 (2)	0.041 (2)	0.0138 (18)	0.0014 (18)	−0.0049 (18)
C9	0.048 (3)	0.054 (3)	0.050 (3)	0.032 (3)	−0.008 (2)	−0.007 (2)
C10	0.076 (4)	0.056 (3)	0.044 (3)	0.041 (3)	−0.005 (3)	0.001 (3)
C11	0.060 (3)	0.042 (3)	0.040 (3)	0.020 (3)	0.009 (2)	0.009 (2)
C12	0.039 (2)	0.036 (2)	0.037 (2)	0.014 (2)	0.0032 (19)	−0.0011 (18)

### Geometric parameters (Å, °)

**Table:**

I1—C1	2.092 (6)	C4—H4	0.93
S1—N1	1.696 (6)	C5—C6	1.352 (10)
S1—C7	1.781 (5)	C5—H5	0.93
N1—C2	1.441 (7)	C6—H6	0.93
N1—H1	0.86	C7—C12	1.390 (7)
N2—O1	1.216 (8)	C7—C8	1.399 (7)
N2—O2	1.219 (7)	C8—C9	1.392 (8)
N2—C8	1.458 (7)	C9—C10	1.365 (10)
C1—C6	1.391 (9)	C9—H9	0.93
C1—C2	1.402 (8)	C10—C11	1.386 (10)
C2—C3	1.392 (8)	C10—H10	0.93
C3—C4	1.375 (9)	C11—C12	1.387 (8)
C3—H3	0.93	C11—H11	0.93
C4—C5	1.400 (11)	C12—H12	0.93
			
N1—S1—C7	102.9 (3)	C5—C6—C1	120.3 (6)
C2—N1—S1	122.0 (5)	C5—C6—H6	119.8
C2—N1—H1	119	C1—C6—H6	119.8
S1—N1—H1	119	C12—C7—C8	116.5 (5)
O1—N2—O2	123.7 (6)	C12—C7—S1	120.5 (4)
O1—N2—C8	117.8 (5)	C8—C7—S1	122.9 (4)
O2—N2—C8	118.5 (6)	C9—C8—C7	121.8 (5)
C6—C1—C2	121.3 (6)	C9—C8—N2	118.4 (5)
C6—C1—I1	119.6 (5)	C7—C8—N2	119.7 (5)
C2—C1—I1	119.1 (4)	C10—C9—C8	120.5 (5)
C3—C2—C1	117.1 (5)	C10—C9—H9	119.8
C3—C2—N1	122.3 (5)	C8—C9—H9	119.8
C1—C2—N1	120.6 (5)	C9—C10—C11	118.8 (6)
C4—C3—C2	121.6 (6)	C9—C10—H10	120.6
C4—C3—H3	119.2	C11—C10—H10	120.6
C2—C3—H3	119.2	C10—C11—C12	120.8 (5)
C3—C4—C5	119.9 (6)	C10—C11—H11	119.6
C3—C4—H4	120.1	C12—C11—H11	119.6
C5—C4—H4	120.1	C11—C12—C7	121.5 (5)
C6—C5—C4	119.8 (6)	C11—C12—H12	119.3
C6—C5—H5	120.1	C7—C12—H12	119.3
C4—C5—H5	120.1		
			
C7—S1—N1—C2	83.9 (5)	C12—C7—C8—C9	1.0 (7)
C6—C1—C2—C3	1.3 (8)	S1—C7—C8—C9	178.9 (4)
I1—C1—C2—C3	−179.0 (4)	C12—C7—C8—N2	180.0 (5)
C6—C1—C2—N1	−179.0 (5)	S1—C7—C8—N2	−2.1 (7)
I1—C1—C2—N1	0.8 (7)	O1—N2—C8—C9	170.0 (6)
S1—N1—C2—C3	−16.1 (8)	O2—N2—C8—C9	−8.4 (8)
S1—N1—C2—C1	164.2 (4)	O1—N2—C8—C7	−9.0 (8)
C1—C2—C3—C4	−0.2 (9)	O2—N2—C8—C7	172.6 (6)
N1—C2—C3—C4	−179.9 (6)	C7—C8—C9—C10	1.0 (9)
C2—C3—C4—C5	−1.2 (10)	N2—C8—C9—C10	−178.0 (5)
C3—C4—C5—C6	1.4 (10)	C8—C9—C10—C11	−2.5 (9)
C4—C5—C6—C1	−0.3 (9)	C9—C10—C11—C12	2.2 (9)
C2—C1—C6—C5	−1.0 (9)	C10—C11—C12—C7	−0.3 (9)
I1—C1—C6—C5	179.2 (5)	C8—C7—C12—C11	−1.3 (7)
N1—S1—C7—C12	1.6 (5)	S1—C7—C12—C11	−179.3 (4)
N1—S1—C7—C8	−176.3 (4)		

### Hydrogen-bond geometry (Å, °)

**Table:**

*D*—H···*A*	*D*—H	H···*A*	*D*···*A*	*D*—H···*A*
C10—H10···O1^i^	0.93	2.55	3.445 (10)	161

Symmetry codes: (i) −*y*+2/3, *x*−*y*−2/3, *z*−2/3.
